# Neuronal activity disrupts myelinated axon integrity in the absence of NKCC1b

**DOI:** 10.1083/jcb.201909022

**Published:** 2020-05-04

**Authors:** Katy L.H. Marshall-Phelps, Linde Kegel, Marion Baraban, Torben Ruhwedel, Rafael G. Almeida, Maria Rubio-Brotons, Anna Klingseisen, Silvia K. Benito-Kwiecinski, Jason J. Early, Jenea M. Bin, Daumante Suminaite, Matthew R. Livesey, Wiebke Möbius, Richard J. Poole, David A. Lyons

**Affiliations:** 1 Centre for Discovery Brain Sciences, University of Edinburgh, Edinburgh, UK; 2 Electron Microscopy Core Unit, Department of Neurogenetics, Max Planck Institute of Experimental Medicine, Göttingen, Germany; 3 Department of Cell and Developmental Biology, University College London, London, UK

## Abstract

Through a genetic screen in zebrafish, we identified a mutant with disruption to myelin in both the CNS and PNS caused by a mutation in a previously uncharacterized gene, *slc12a2b,* predicted to encode a Na^+^, K^+^, and Cl^−^ (NKCC) cotransporter, NKCC1b. *slc12a2b/*NKCC1b mutants exhibited a severe and progressive pathology in the PNS, characterized by dysmyelination and swelling of the periaxonal space at the axon–myelin interface. Cell-type–specific loss of *slc12a2b/*NKCC1b in either neurons or myelinating Schwann cells recapitulated these pathologies. Given that NKCC1 is critical for ion homeostasis, we asked whether the disruption to myelinated axons in *slc12a2b/*NKCC1b mutants is affected by neuronal activity. Strikingly, we found that blocking neuronal activity completely prevented and could even rescue the pathology in *slc12a2b/*NKCC1b mutants. Together, our data indicate that NKCC1b is required to maintain neuronal activity–related solute homeostasis at the axon–myelin interface, and the integrity of myelinated axons.

## Teaser

Marshall-Phelps, Kegel, et al. find that disruption to the solute cotransporter NKCC1 greatly dysregulates myelin and the periaxonal space of peripheral nerves. Neurons and myelinating glia both require NKCC1 to maintain homeostasis following neuronal activity, identifying it as a critical regulator of myelinated axon physiology.

## Introduction

Interactions between axons and myelinating glia (Schwann cells in the peripheral nervous system [PNS] and oligodendrocytes in the central nervous system [CNS]) underpin many aspects of nervous system formation, health, and function. The myelination of axons has long been known to facilitate rapid saltatory conduction due to the multilamellar wrapping of myelin sheaths around axons and the sequestration of distinct ion channels to discrete domains along the myelinated axon, including voltage-gated Na^+^ channels to nodes of Ranvier. Recently, however, it has become clear that the physiology of myelinated axons is more complex than previously thought. For example, it has been proposed that electrical conduction through the periaxonal space between the axon and the overlying myelin sheath also contributes to the fundamental nature of conduction along myelinated axons (Cohen et al., 2020). Indeed, numerous ion channels, transporters, and neurotransmitter receptors are present at the axon–myelin interface and are thus well placed to regulate myelinated axon physiology, structure, and integrity (Suminaite et al., 2019). These include axonal ion channels, including potassium (K^+^) channels, that extrude ions into the periaxonal space upon neuronal activity and channels in the juxtaposed myelin sheath that buffer ions, including K^+^, from the periaxonal space (Rash et al., 2016; Wang et al., 1993; Schirmer et al., 2018; Larson et al., 2018). In addition, monocarboxylate transporters juxtaposed at the axon–myelin interface are thought to mediate the transfer of metabolic substrates from the myelinating oligodendrocyte to the axon through the periaxonal space (Saab et al., 2016; Fünfschilling et al., 2012). Furthermore, receptors for neurotransmitters, neuromodulators, and neuropeptides are localized to the axon–myelin interface (Saab et al., 2016; Micu et al., 2016, 2018) and have been proposed to mediate adaptations to myelin structure in response to neuronal activity that may even fine-tune neural circuit function (Almeida and Lyons, 2017; Monje, 2018; Fields, 2015).

The importance of physiological interactions between axons and myelinating glia at the axon–myelin interface is only now becoming clear, with dysregulation implicated in susceptibility to seizures (Larson et al., 2018), neuronal health (Schirmer et al., 2018; Saab et al., 2016; Lee et al., 2012; Jha et al., 2020), and potentially also neurodevelopmental, neuropsychiatric, and neurodegenerative disorders (Micu et al., 2018; Gibson et al., 2018; Stassart et al., 2018). To gain deeper insight into myelinated axon biology, we executed a gene discovery screen in zebrafish, through which we identified a mutant with severe disruption to myelin. This phenotype was caused by a mutation in a previously uncharacterized zebrafish gene, *slc12a2b*, predicted to encode a sodium (Na^+^), potassium (K^+^), and chloride (Cl^−^) cotransporter, NKCC1b. NKCC1 cotransports Na^+^, K^+^, and Cl^−^ together with water, typically from the extracellular space into cells (Zeuthen and MacAulay, 2012; MacAulay and Zeuthen, 2010), and has been implicated in regulating many aspects of ion and fluid homeostasis in the healthy nervous system (Delpire et al., 1999; Flagella et al., 1999; Dixon et al., 1999; MacVicar et al., 2002; Su et al., 2002; Larsen et al., 2014; Stenesen et al., 2019) and following injury and disease (Blaesse et al., 2009; Ben-Ari, 2017; Yousuf et al., 2017; Gagnon and Delpire, 2013). Here, we show that NKCC1b is required to maintain the integrity of myelinated axons following neuronal activity, implicating it as a key regulator of solute homeostasis at the axon–myelin interface.

## Results and discussion

### Mutation of zebrafish slc12a2b disrupts myelination

**Figure 1.**
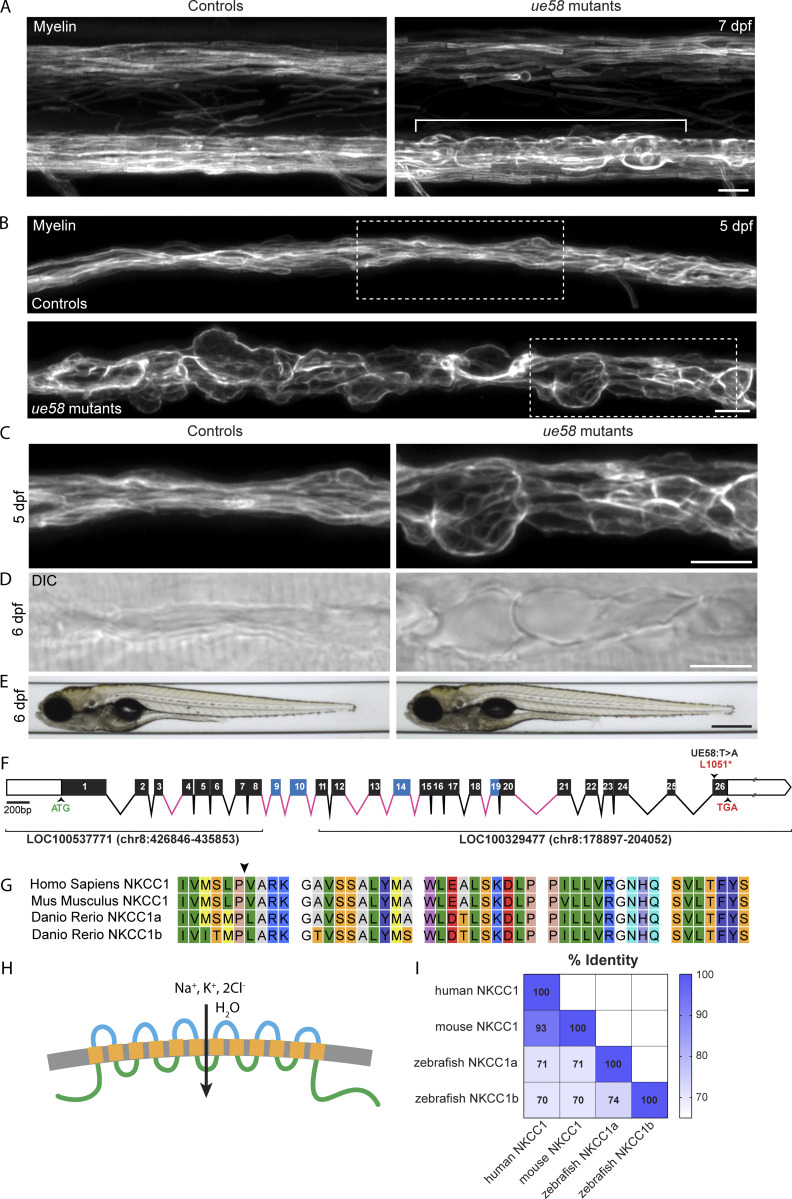
***ue58* mutant zebrafish have a severe, peripheral nerve myelin pathology. (A)** Confocal images of the spinal cord of Tg(mbp:EGFP-CAAX) control (left) and *ue58* mutant (right) at 7 dpf showing disruption to CNS myelin (region within brackets). Scale bar, 10 µm. **(B)** Confocal images of the pLLn in Tg(mbp:EGFP-CAAX) control (top) and *ue58* mutant (bottom) animals at 5 dpf showing major disruption to myelin. Scale bar, 10 µm. **(C)** Higher magnification images of areas demarcated in B showing myelin in control (left) and *ue58* mutant (right) animals. Scale bar, 10 µm. **(D)** DIC images of Tg(mbp:EGFP-CAAX) control (left) and *ue58* mutants (right) at 6 dpf showing appearance of tissue edema. Scale bar, 10 µm. **(E)** Brightfield images of control (left) and *ue58* mutants (right) at 6 dpf showing generally normal morphological development. Scale bar, 0.5 mm. **(F)** Genomic structure of the zebrafish *slc12a2b* gene, showing exons (boxes) and introns (lines). White boxes denote untranslated regions. Exons in black were annotated in partial genomic sequences LOC100537771 and LOC100329477 and matched homologous exons in the orthologue *slc12a2a*. Exons in blue did not align with any annotated genomic sequence, and their limits were inferred by homology with *slc12a2a* genomic structure. Exons are drawn to scale relative to each other; introns in pink contain unknown bases (N) and are of unknown size. The start (ATG) and stop (TGA) codons are indicated in green and red, respectively. The *ue58* allele has a T>A mutation in exon 26 leading to a premature stop codon. **(G)** Alignment of the 40 most C-terminal amino acids of NKCC1b shows high similarity between species in this domain. Arrowhead indicates the position of the premature stop codon introduced by *ue58*. **(H)** Protein structural prediction algorithms, using CCTOP, indicate that NKCC1b in zebrafish is likely to have intracellular N and C termini and 12 transmembrane domains. **(I)** Sequence similarities of the protein products of zebrafish NKCC1a, NKCC1b, and murine and human NKCC1 homologues.

**Figure S1.**
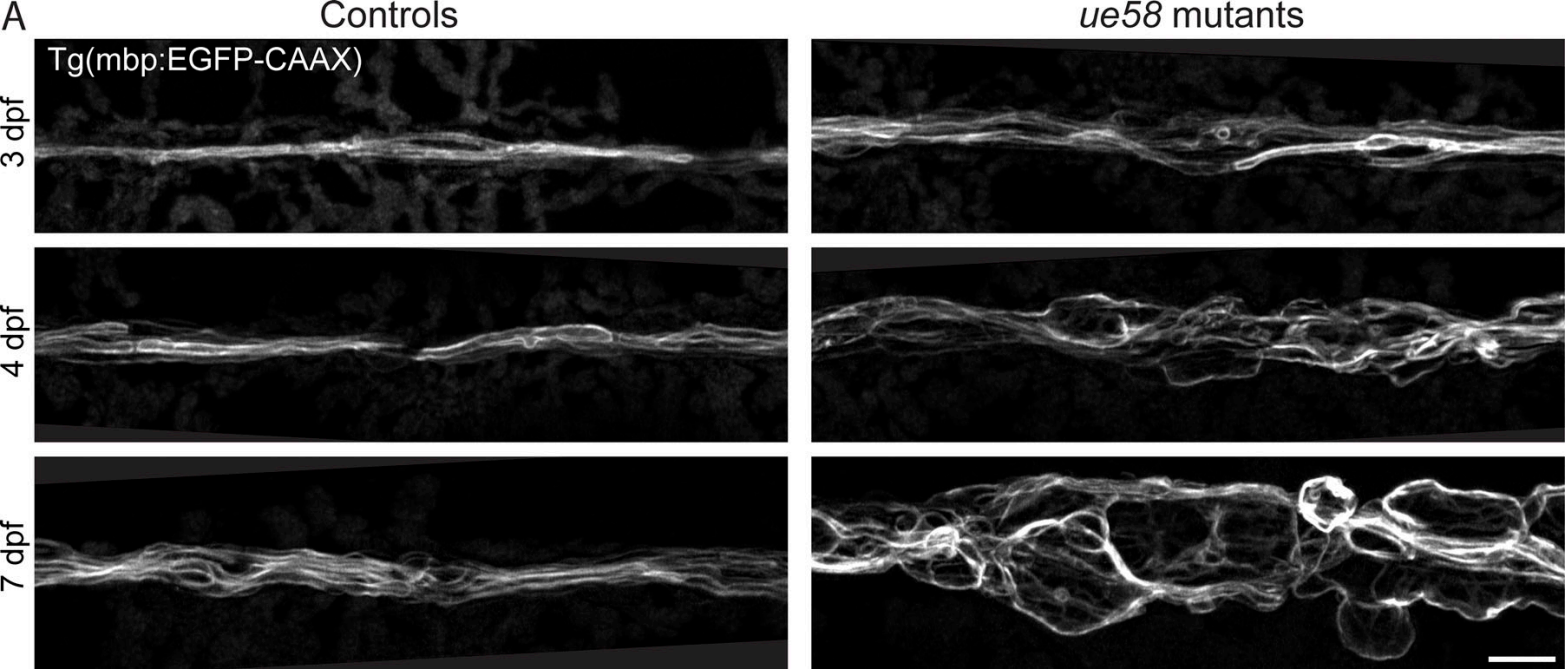
**Myelin in *ue58* mutants forms normally but becomes progressively disrupted. (A)** Confocal images of the pLLn in Tg(mbp:EGFP-CAAX) control heterozygote (left) and *ue58* homozygote mutant (right) animals at 3, 4, and 7 dpf. Scale bar, 10 µm.

To help elucidate mechanisms underpinning myelinated axon formation, health, and function, we performed a mutagenesis-based gene discovery screen using zebrafish (Kegel et al., 2019; Klingseisen et al., 2019; see Materials and methods). To assess myelin, we used the transgenic reporter Tg(mbp:EGFP-CAAX) in which green fluorescent protein is targeted to the membrane of myelinating glia (Almeida et al., 2011). One of the mutant alleles that we identified, *ue58*, exhibited a striking phenotype, whereby myelin was disrupted in both the PNS and CNS (Fig. 1 A; Moyon et al., 2019* Preprint*). Although CNS pathology was evident in *ue58* mutants, disruption to myelin in the PNS was more prominent and emerged earlier. We found that myelin made by Schwann cells along the posterior lateral line nerve (pLLn) was particularly disrupted (Fig. 1, B and C). In addition, differential interference contrast (DIC) imaging of *ue58* mutants revealed extensive edema (excess fluid) along the entire length of the pLLn of all mutants (Fig. 1 D). Time-course analyses indicated that myelin appears to form relatively normally by 3 d postfertilization (dpf) in *ue58* mutants but become progressively disrupted from 4 dpf onwards (Fig. S1). Despite the severe derangement of myelin and extensive nerve edema, *ue58* homozygous mutants are viable and have no other overt developmental disruption (Fig. 1 E).

**Figure S2.**
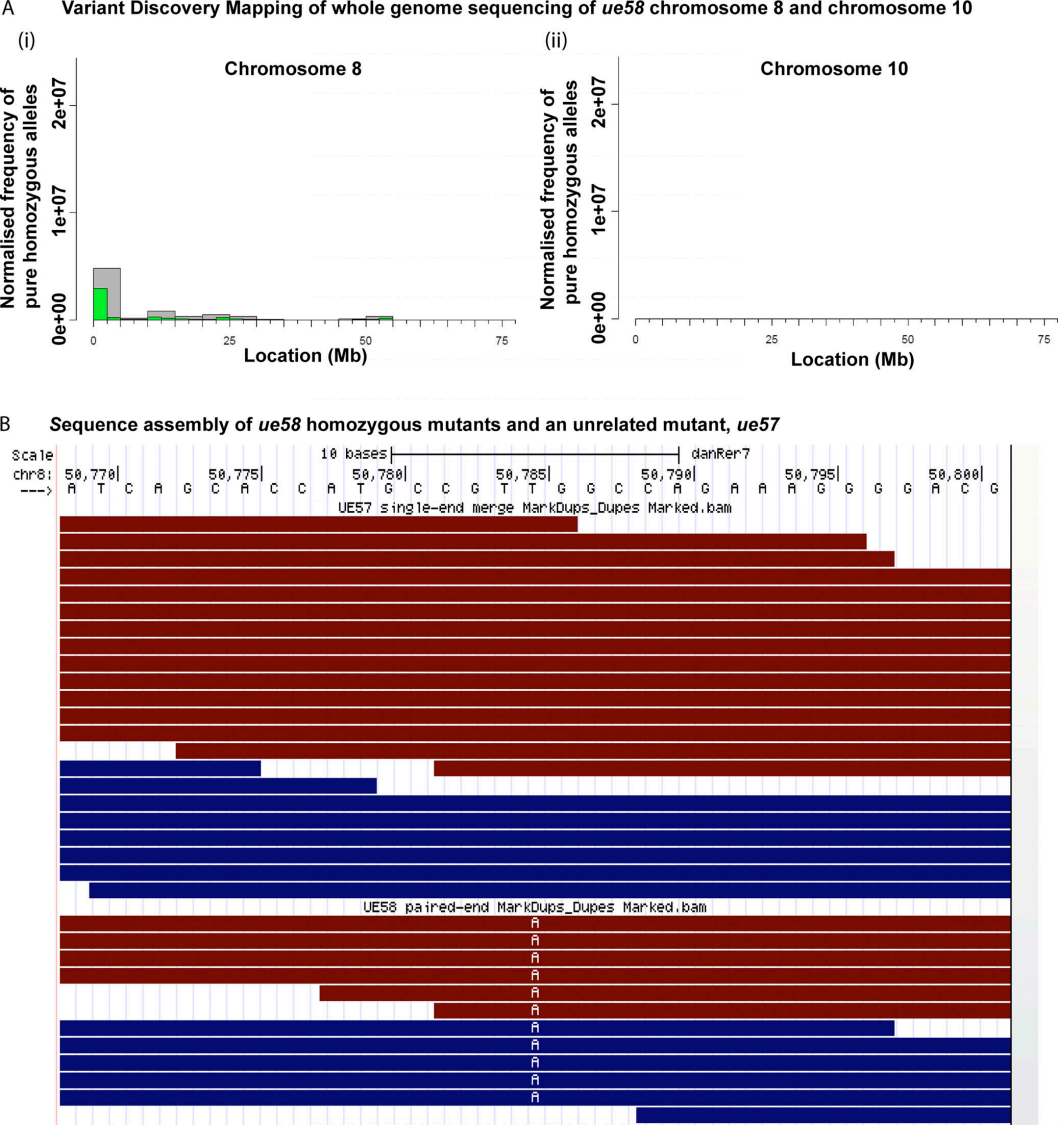
**Molecular characterization of the *ue58* mutation and *slc12a2b*. (A)** Variant discovery mapping plots from the CloudMap pipeline showing the normalized frequency of pure homozygous variants (allele frequency in recombinant pool = 1.0) in 2.5-Mb (green) or 5-Mb (gray) bins. Note linkage at the beginning of chromosome 8, but none in 10, as a comparison, and where *slc12a2a* is localized. **(B)** Raw sequence reads in the candidate region defined by mapping shows a T to A change in the *ue58* mutant reads, but not in an unrelated mutant, *ue57*.

To identify the mutation responsible for the *ue58* phenotype, we performed whole-genome sequencing of mutant larvae (Materials and methods). This identified genetic linkage between the mutant phenotype and the start of chromosome 8 (Fig. S2), wherein we identified a T to A base pair change predicted to induce a stop codon in an ORF partially annotated at the time of sequence analysis (Fig. 1 F and Fig. S2; Materials and methods). We identified sequence similarity between this partially annotated region and another zebrafish gene, *slc12a2* (Abbas and Whitfield, 2009), which encodes an NKCC1 cotransporter (Chew et al., 2019), to which we found no linkage of the mutant phenotype (Fig. S2). To further characterize the candidate gene on chromosome 8, we amplified mRNA based on the partially annotated sequence, and identified a product similar to that encoded by the previously defined *slc12a2* gene (Fig. 1, F–I). Alignment of this new NKCC1-like ORF to genomic sequence indicated that the *ue58* mutation introduced a premature stop codon in the last exon (exon 26) of the gene (Fig. 1 F), which is predicted to truncate the last highly conserved 40 amino acids of the protein (Fig. 1 G).

**Figure S3.**
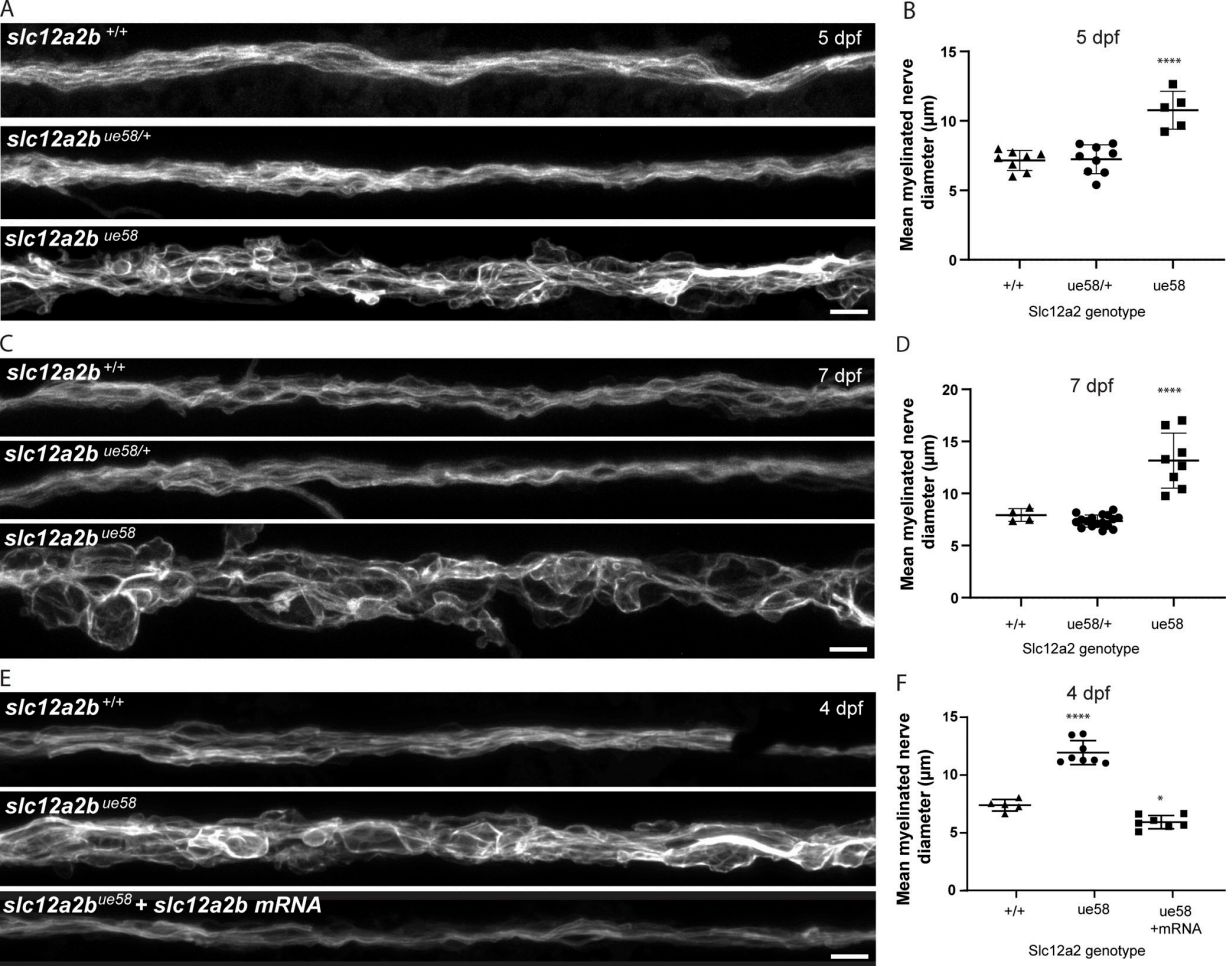
**Disruption to *slc12a2b* leads to myelin pathology. (A)** Images of the pLLn in Tg(mbp:EGFP-CAAX) wild-type, *slc12a2b ^ue58/+^* and *slc12a2b* mutant animals at 5 dpf. Scale bar, 10 µm. **(B)** Quantitation of mean myelinated nerve diameter in wild-type, *slc12a2b ^ue58/+^* and *slc12a2b* mutant animals at 5 dpf (wild type 7.2 ± 0.7 µm vs. *slc12a2b ^ue58/+^* 7.2 ± 1 µm vs. *slc12a2b ^ue58^* 10.8 ± 1.4 µm). Error bars represent mean ± SD. One-way ANOVA followed by Tukey’s multiple comparison test was used to assess statistical significance (ANOVA *F*(2,19) = 23.9, P < 0.0001). Each point represents an individual animal. ****, P < 0.0001. **(C)** Images of Tg(mbp:EGFP-CAAX) wild-type, *slc12a2b ^ue58/+^*, and *slc12a2b* mutant animals at 7 dpf. Scale bar, 10 µm. **(D)** Quantitation of mean myelinated nerve diameter in wild-type, *slc12a2b ^ue58/+^* and *slc12a2b* mutant animals at 7 dpf (wild type 7.9 ± 0.6 µm vs. *slc12a2b ^ue58/+^* 7.4 ± 0.6 µm vs. *slc12a2b ^ue58^* 13.2 ± 2.6 µm). Error bars represent mean ± SD. One-way ANOVA followed by Tukey’s multiple comparison test was used to assess statistical significance (ANOVA *F*(2,26) = 44.1, P < 0.0001). Each point represents an individual animal. ****, P < 0.0001. **(E)** Images of Tg(mbp:EGFP-CAAX) wild-type, control-injected *slc12a2b* mutant, and *slc12a2b* mRNA–injected mutant animals at 4 dpf. Scale bar, 10 µm. **(F)** Quantitation of mean myelinated nerve diameter in wild-type, control-injected *slc12a2b* mutant, and *slc12a2b* mRNA–injected mutant animals at 4 dpf (wild type 6.7 ± 0.5 µm vs. *slc12a2b ^ue58^* 11.2 ± 1 µm vs. *slc12a2b^ue58^* + mRNA 5.2 ± 0.6 µm). Error bars represent mean ± SD. One-way ANOVA followed by Tukey’s multiple comparison test was used to assess statistical significance (ANOVA *F*(2,17) = 1.2, P < 0.0001). Each point represents an individual animal. *, P < 0.05; ****, P < 0.0001.

**Figure S4.**
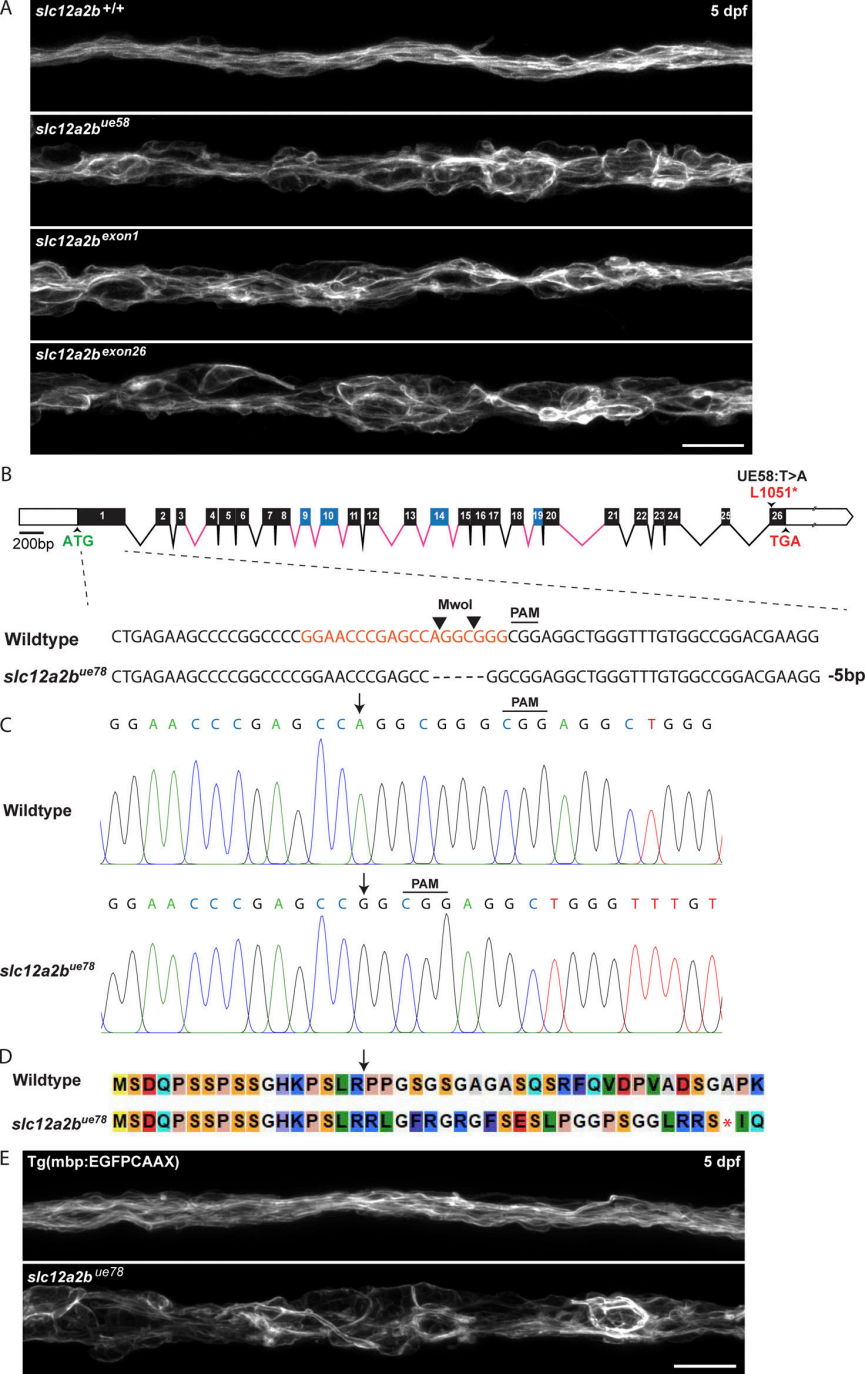
**Validation of *slc12a2b* loss of function in CRISPR/Cas9 mutant animals. (A)** Images of the pLLn in Tg(mbp:EGFP-CAAX) wild-type (top), *slc12a2b* mutant animals (second panel) and Tg(mbp:EGFP-CAAX) wild-type animals injected with CRISPR guide RNAs targeting exon 1 of *slc12a2b* (third panel) and exon 26 (bottom panel) of *slc12a2b*. Scale bar, 20 µm. **(B)** Genomic structure of the zebrafish *slc12a2b* gene, as in Fig. 1, showing the CRISPR guide RNA targeting sequence located within exon 1 (orange). Sanger sequencing from second generation *slc12a2b^ue78^* mutants reveals a 5 bp deletion upstream of the PAM cleavage site within exon 1 of the *slc12a2b* gene. Deletion of this sequence disrupts the MwoI restriction enzyme recognition site, allowing mutant animals to be genotyped based on the presence of undigested product. **(C) **Sanger sequence chromatograms of *slc12a2b^ue78^* mutants compared with wild-type controls showing region of 5 bp deletion (arrows). **(D)** Alignment of predicted amino acid sequences in *slc12a2b^ue78^* mutants and wild-type controls. The 5 bp deletion in mutants produces a frameshift in the coding sequence (arrow) and introduction of a premature translational stop codon (asterisk). **(E)** Confocal images of 5 dpf Tg(mbp:EGFP-CAAX) wild-type (top) and second-generation *slc12a2b^ue78^* mutant animals (bottom) showing major myelin disruption along the pLLn. Scale bar, 20 µm.

Quantitative analyses of the myelin phenotype indicated that only animals homozygous for the *ue58* mutation exhibited a significant disruption to myelination, with heterozygous animals appearing similar to wild-type animals (Fig. S3, A–D). To further test whether the *ue58* mutant phenotype was indeed due to disruption of this putative NKCC1-encoding gene, we injected synthetic mRNA encoding our newly isolated NKCC1-like product into *ue58* mutants and found that this rescued their myelin defects (Fig. S3, E and F). Given the incomplete annotation of the genome of chromosome 8 at the mutant-linked locus harboring the NKCC1-like sequence, we independently targeted two regions of the candidate gene using CRISPR guide RNAs. Independent targeting of exon 1 or exon 26, where the causative mutation resided, resulted in severe disruption to myelin morphology, as assessed by Tg(mbp:EGFP-CAAX) (Fig. S4). Together our data indicate that a novel gene encoding an NKCC1-like protein is required for the maintenance of myelin and that its C terminus is functionally essential. Given the previous characterization of a separate NKCC1-encoding gene (*slc12a2*) in zebrafish (Abbas and Whitfield, 2009), we designate our newly described gene as *slc12a2b* and the encoded protein as NKCC1b and suggest that the originally annotated gene be referred to as *slc12a2a* and its encoded protein as NKCC1a. The crystal structure for the zebrafish NKCC1a protein was recently solved and found similar to that of mouse and human NKCC1 (Chew et al., 2019). NKCC1a and NKCC1b have the same predicted structure (Fig. 1 H) and degree of similarity to their mouse and human NKCC1 counterparts (Fig. 1 I), which further indicates that *slc12a2b* encodes an NKCC1 cotransporter.

### Disruption to NKCC1b leads to enlargement of the periaxonal space and dysmyelination

Given that NKCC1 typically cotransports ions (Na^+^, K^+^, and 2Cl^−^) and water into cells, loss of its function would be predicted to lead to extracellular ion and water accumulation, which could account for the observed edema and dysregulation of myelin seen in the *slc12a2b^ue58^* mutant. In line with this, glial-specific disruption to an orthologue of NKCC1 (Ncc69) has been shown to lead to fluid accumulation in the extracellular space of peripheral nerves in *Drosophila* (Leiserson et al., 2011). We have recently shown, using immunogold labeling of an anti-NKCC1 antibody visualized by EM, that NKCC1 is localized at the axon–myelin interface in the mammalian CNS, both in the innermost layer of the myelin sheath and the juxtaposed axon itself (Moyon et al., 2019* Preprint*). This positions NKCC1 as a potential regulator of ion and solute homeostasis at the axon–myelin interface of vertebrate myelinated axons.

**Figure 2.**
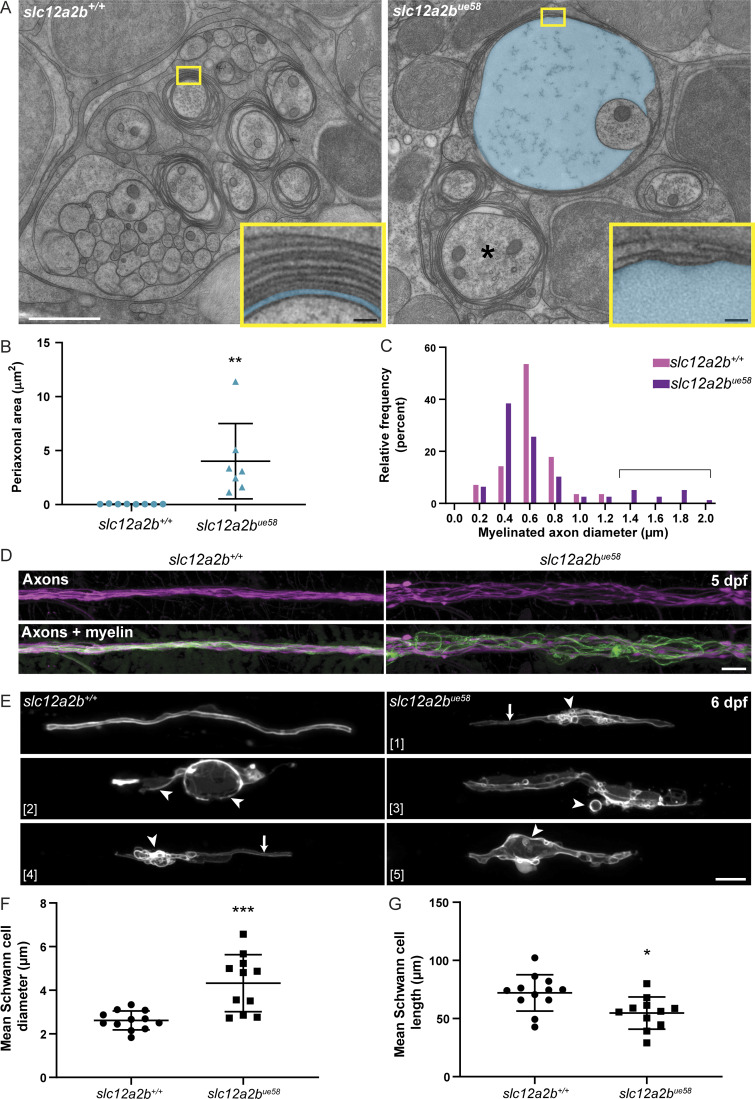
**Disruption to NKCC1b leads to swelling of the periaxonal space, dysmyelination, and axonal disorganization. (A)** Electron micrographs of high-pressure–frozen pLLn in control (left) and *slc12a2b^ue58^* mutant (right) at 5 dpf. *slc12a2b^ue58^* mutants show significant enlargement of the periaxonal space, highlighted in blue and enlarged axons (asterisk). Insets show a higher magnification to highlight the periaxonal space in controls and *slc12a2b^ue58^* mutants. White scale bar, 1 µm. Black scale bars, 50 nm. **(B) **Quantification of periaxonal area in control and *slc12a2b^ue58^* mutants (control 0.05 ± 0.02 µm^2^ vs. *slc12a2b^ue58^* 4 ± 3.5 µm^2^, P = 0.0065). Error bars represent mean ± SD. A two-tailed Student’s *t* test was used to assess statistical significance. Each point represents an individual myelinated axon from three control and five *slc12a2b^ue58^* mutant animals. **, P < 0.01. **(C)** Quantification of the diameter of myelinated axons in control and *slc12a2b^ue58^* mutants. Bracket indicates axons in the mutant with greater than normal diameter. **(D)** Confocal images of live Tg(cntn1b:mCherry), Tg(mbp:EGFP-CAAX) double-transgenic control (left) and *slc12a2b^ue58^* mutant (right) animals at 5 dpf indicates axonal defasciculation and derangement of myelin. Scale bar, 10 µm. **(E)** Confocal images of individual mosaically labeled Schwann cells in control (top left panel) and *slc12a2b^ue58^* mutants (panels 1–5) highlighting the variable morphological manifestation of the mutant phenotype. Scale bar, 10 µm. Arrows point to regions of normal appearing myelin and arrowheads to dysmyelination. **(F)** Quantitation of mean Schwann cell diameter in maximum intensity projection images of single Schwann cells at 6 dpf (control 2.6 ± 0.4 µm vs. *slc12a2b^ue58^* 4.3 ± 1.3 µm, P = 0.0003). Error bars represent mean ± SD. A two-tailed Student’s *t* test was used to assess statistical significance. Each point represents a single cell from 11 control and 10 *slc12a2b^ue58^* mutant animals. Scale bar, 10 µm. ***, P < 0.001. **(G)** Quantitation of mean Schwann cell length in maximum intensity projection images of single Schwann cells at 6 dpf (control 72.1 ± 15.7 µm vs. *slc12a2b^ue58^* 54.7 ± 13.8 µm, P = 0.011). Error bars represent mean ± SD. A two-tailed Student’s *t* test was used to assess statistical significance. Each point represents a single cell from 11 control and 10 *slc12a2b^ue58^* mutant animals. *, P < 0.05.

To test how NKCC1b disruption affects peripheral nerve ultrastructure, we performed high-pressure freezing–based cryopreservation of zebrafish larvae for transmission EM (TEM; Weil et al., 2019). To increase the likelihood of optimally preserving tissue with edema, we cryopreserved animals between 4 and 5 dpf, when the phenotype first emerged. Because we performed our TEM analyses at the onset of pathology, we observed that many axons were ensheathed by normal appearing myelin, corroborating our reporter-based time course analyses, which showed that myelin first appears to be relatively normal before pathology progresses over time. However, our TEM analyses did reveal striking edema in the periaxonal space between axons and overlying multilamellar myelin sheaths in six out of eight mutant nerves examined (Fig. 2 A). In sufficiently well-preserved cases, we measured the periaxonal space between the axon and the first layer of myelin and found that that this was substantially enlarged in mutant axons (Fig. 2 B). Edema was always found in association with myelinated axons and never in other regions of the nerve, including unmyelinated axon bundles, in or near Schwann cell bodies, or in neighboring tissue. Our observations support the premise that NKCC1 regulates solute homeostasis at the axon–myelin interface.

**Figure S5.**
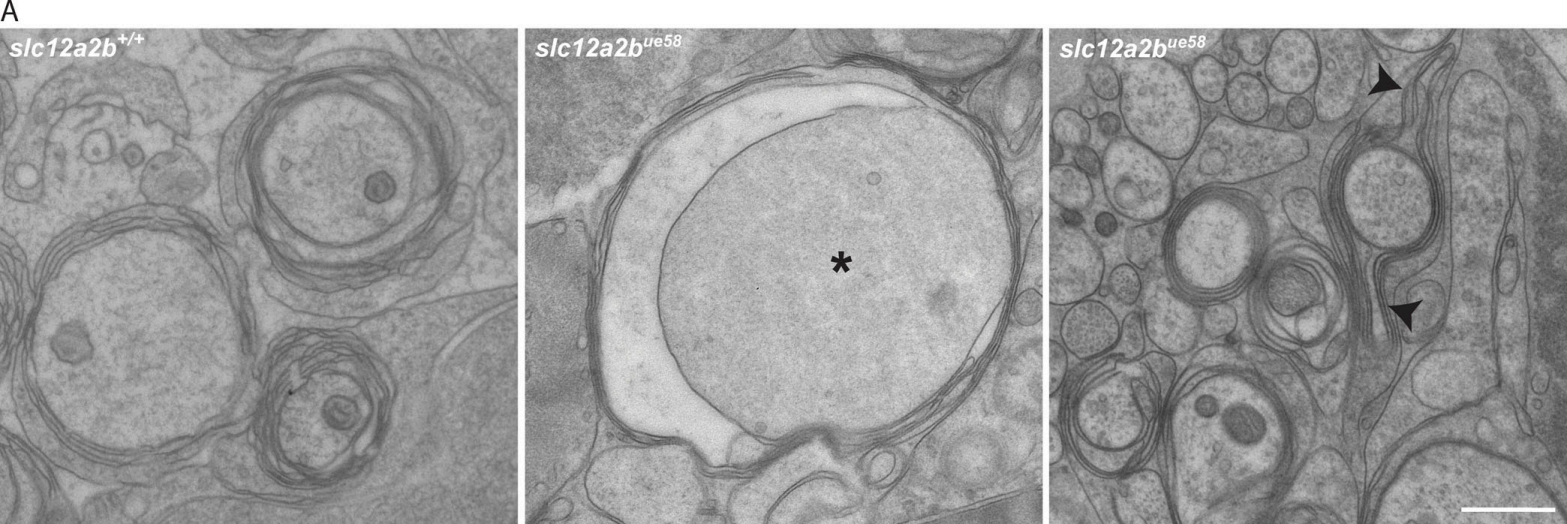
**Periaxonal space swelling, axonal enlargement, and myelin outfoldings in *slc12a2b^ue58^* mutants. (A)** TEM images of the pLLn in control (left) and *slc12a2b^ue58^* mutant animals showing an enlarged axon (asterisk) with periaxonal swelling (middle) and myelin outfoldings (arrowheads; right). Scale bar, 1 µm.

Our TEM analysis also revealed evidence of occasional myelin outfoldings (Fig. S5) and a small number of abnormally large axons (Fig. 2, A and C; and Fig. S5), suggesting the possibility of additional pathologies in *slc12a2b* mutants. To investigate this further, we analyzed transgenic reporters that allowed us to more broadly assess axonal and myelinating Schwann cell morphology. To visualize axons in the context of myelination, we imaged the double transgenic reporter Tg(cntn1b:mCherry, mbp:EGFP-CAAX) (Fig. 2 D). This showed that axons of the pLLn were defasciculated and had localized swelling at discrete points along their length (Fig. 2 D), likely explaining the appearance of occasional enlarged axons noted by TEM. To assess Schwann cell morphology, we mosaically labeled cells using a membrane-tethered reporter (Materials and methods) and observed individual mutant Schwann cells with variable levels of disruption (Fig. 2 E). While many *slc12a2b^ue58^* mutant Schwann cells exhibited signs of significant swelling, this was accompanied by a shortening of cell length compared with controls (Fig. 2, E–G). We also observed that cells could undergo significant membrane blebbing, while others had myelin outfoldings as indicated by our TEM analysis (Fig. 2 E and Fig. S5). In some cases, we saw individual Schwann cells with both areas of normal appearing myelin and grossly disrupted morphology (Fig. 2 E), indicating the dynamic, progressive nature of the NKCC1b loss of function pathology.

Together our observations show that disruption to NKCC1b leads to major dysregulation of the periaxonal space and the integrity of myelinated axons. It remains to be determined whether the distinct morphological manifestations of NKCC1b loss of function all reflect dysregulation of ion and fluid homeostasis at the axon–myelin interface or multiple distinct roles for NKCC1b in axons and/or myelinating glia.

### Loss of NKCC1b function in myelinating glia or neurons disrupts myelinated axon integrity

**Figure 3.**
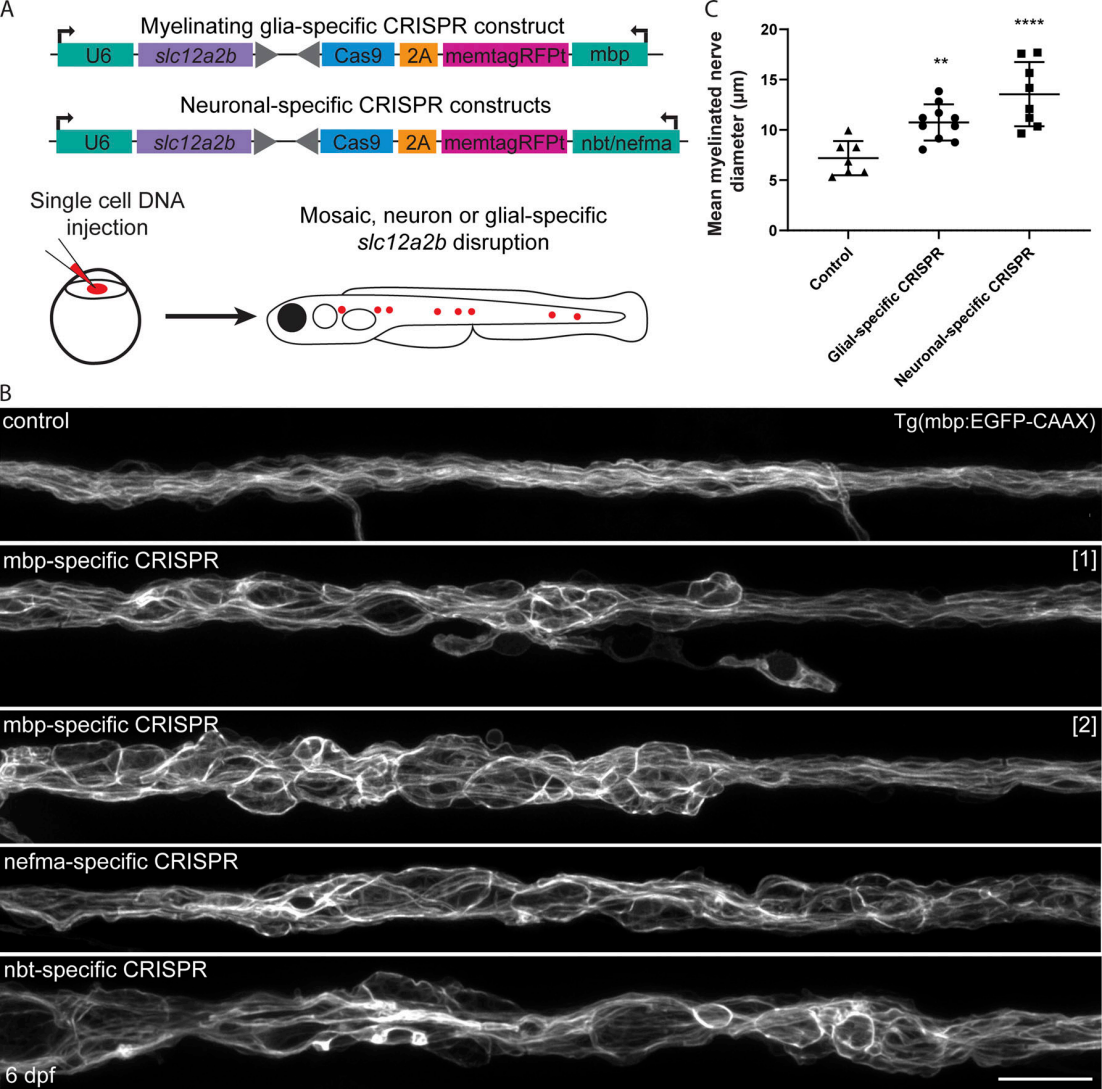
**Cell-type–specific disruption of *slc12a2b* in either neurons or Schwann cells leads to myelin pathology. (A)** Schematic overviews of constructs used to induce *slc12a2b* mutations in myelinating glial cells (top) and neurons (bottom), which are separately injected into embryos at the single-cell stage, leading to mosaic expression (red dots) at later stages, when myelination is examined. **(B)** Confocal images of Schwann cells along the pLLn in a 6 dpf Tg(mbp:EGFP-CAAX) control (top), two genetically mosaic animals in which *slc12a2b* has been targeted in myelinating glial cells, and two further mosaic animals in which *slc12a2b* has been targeted in neurons. Scale bar, 20 µm. **(C)** Quantitation of mean myelinated nerve diameter in controls compared with larvae with glial- or neuronal-specific *slc12a2b*-specific disruption at 6 dpf (control 7.2 ± 1.7 µm vs. glial-specific *slc12a2b* disruption 10.7 ± 1.8 µm vs. neuronal-specific *slc12a2b* disruption 13.6 ± 3.2 µm). Error bars represent mean ± SD. One-way ANOVA followed by Tukey’s multiple comparison test was used to assess statistical significance (ANOVA *F*(2,22) = 14.09, P = 0.0001). Each point represents an individual animal. **, P < 0.01; ****, P < 0.0001.

To test whether NKCC1b mediates distinct and/or overlapping roles in myelinating Schwann cells and neurons, we undertook cell-type–specific approaches using CRISPR-Cas9 technology to target *slc12a2b* function. To do so, we placed a gRNA targeting exon 1 of *slc12a2b* in a plasmid that also drove expression of the Cas9 nuclease in a cell-type–specific manner. To drive expression in myelinating glia, we used the myelin basic protein (mbp) gene regulatory sequence and for neurons either nefma or nbt gene regulatory sequences (Fig. 3 A; Materials and methods). We first saw the dysmyelination and edema characteristic of *slc12a2b^ue58^* mutants in animals in which *slc12a2b* was specifically targeted in myelinating glia (Fig. 3, B and C). Reflecting the mosaic nature of our cell-type–specific targeting, the phenotype in animals with myelinating glial loss of *slc12a2b* function was observed discontinuously along the nerve. We also observed disruption to myelin morphology upon neuron-specific targeting of *slc12a2b* function (Fig. 3, B and C). Reflecting the long-range axonal projections of individual neurons of the posterior lateral line ganglion, we saw disruption to myelin along the entire length of affected nerves in animals with neuron-specific loss of *slc12a2b* function.

Together, our results indicate that disruption to NKCC1b in either myelinating Schwann cells or neurons is sufficient to drive the pathology seen in constitutive mutants, suggesting that NKCC1 functions at the axon–myelin interface in both the axon and Schwann cell to ensure myelinated axon integrity.

### Neuronal activity drives the myelin pathology observed in NKCC1b mutants

**Figure 4.**
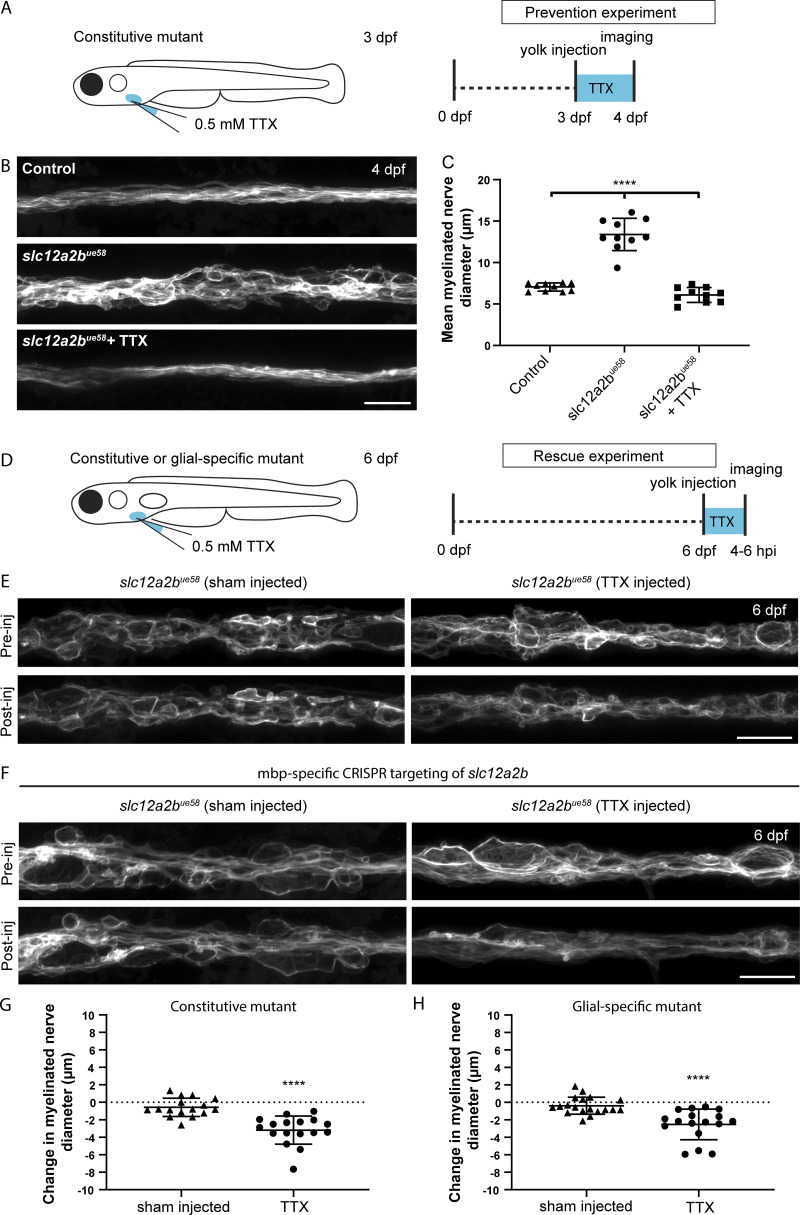
**Neuronal activity drives peripheral nerve pathology in *slc12a2b* mutants. (A)** Schematic overview of when, where and for how long TTX was applied to *slc12a2b^ue58^* mutants. **(B)** Confocal images of a Tg(mbp:EGFP-CAAX) control (top), *slc12a2b^ue58^* mutant (middle), and *slc12a2b^ue58^* mutant injected with TTX (bottom). Scale bar, 20 µm. **(C)** Quantitation of mean myelinated nerve diameter in controls, *slc12a2b^ue58^* mutants and *slc12a2b^ue58^* mutants injected with TTX (control 7.1 ± 0.5 µm vs. *slc12a2b^ue58^* 13.4 ± 1.9 µm vs. *slc12a2b^ue58^*+ TTX 6.1 ± 0.9 µm). Error bars represent mean ± SD. One-way ANOVA followed by Tukey’s multiple comparison test was used to assess statistical significance (ANOVA *F*(2,27) = 97, P < 0.0001). Each point represents an individual animal. ****, P < 0.0001. **(D)** Schematic overview of when, where, and for how long TTX was applied to either constitutive or glial-specific *slc12a2b* mutants. **(E) **Confocal images of 6 dpf *slc12a2b^ue58^* mutant larvae. Top and bottom panels show the same region of the pLLn before and 4–6 h after injection with either a control solution (left), or TTX (right). Scale bar, 20 µm. **(F)** Confocal images of 6 dpf Tg(mbp:EGFP-CAAX) larvae, in which *slc12a2b* has been targeted in myelinating glial cells. Top and bottom panels show the same region of the pLLn before and 4–6 h after injection with either a control solution (left) or TTX (right). Scale bar, 20 µm. **(G)** Quantitation of the change in mean myelinated nerve diameter following injection with either a control solution or TTX in *slc12a2b* mutants (G; sham injected −0.6 ± 1.1 µm vs. TTX −3.2 ± 1.6 µm, P < 0.0001) or animals in which *slc12a2b* has been disrupted specifically in myelinating glial cells (H; sham injected −0.4 ± 1 µm vs. TTX −2.5 ± 1.7 µm, P < 0.0001). Two-tailed Student’s *t* test was used to assess statistical significance. Each point represents an individual animal. ****, P < 0.0001.

The fact that NKCC1 is important for ion homeostasis and that it is localized to the axon–myelin interface (Moyon et al., 2019* Preprint*) suggested the possibility it may have a key role in regulating ion homeostasis in myelinated axons following neuronal activity. To test this hypothesis, we inhibited neuronal activity by injecting tetrodotoxin (TTX) into the yolk of 3 dpf control and constitutive *slc12a2b^ue58^* mutant animals to block action potential firing (Fig. 4 A). We confirmed the efficacy of TTX injections by assessing motility and only pursued analyses of fully paralyzed zebrafish larvae. We found that myelin at 4 dpf was quantitatively indistinguishable between control animals and TTX-injected *slc12a2b^ue58^* mutants, whereas sham-injected mutants exhibited their characteristic myelin pathology (Fig. 4, B and C). This indicates that the pathology seen in animals with loss of NKCC1b function is driven by neuronal activity. We next asked whether the severe disruption to myelinated axons in *slc12a2b^ue58^* mutants might be reversible if neuronal activity was inhibited. We found that TTX injection at 6 dpf, after pathology had emerged, was indeed capable of reducing myelin disruption in *slc12a2b^ue58^* mutants (Fig. 4, D, E, and G), indicating that ongoing neuronal activity contributes to the progression of pathology. We next wanted to test whether *slc12a2b* was required specifically in myelinating glia to maintain myelin integrity in response to neuronal activity. We grew Tg(mbp:EGFP-CAAX) animals in which *slc12a2b* was disrupted specifically in myelinating Schwann cells to 6 dpf and screened them for the presence of myelinated axon pathology. We then injected a subset of animals exhibiting pathology with either vehicle or TTX and assessed myelination (Fig. 4, D and F). While we continued to see myelin pathology in sham-injected animals with myelinating glial-specific targeting of *slc12a2b*, we observed that pathology was significantly attenuated within hours following TTX injection (Fig. 4, F and H). This result indicates that NKCC1b is required by myelinating Schwann cells to maintain solute homeostasis following neuronal activity and myelinated axon integrity.

Our results raise the question as to how neuronal activity can trigger such a severe pathology in myelinated axons in the absence of either neuronal or Schwann cell NKCC1b. We predict that ions released into the periaxonal space upon action potential firing might not be appropriately buffered without NKCC1b and that this leads to a cascade of dysregulation that culminates in the severe pathology observed. Given that K^+^ ions are released into the periaxonal space upon action potential firing, failure to buffer K^+^ may be a key contributor to the observed pathology, but how NKCC1b loss of function leads to ion and solute imbalance causing the observed pathologies (and so rapidly) remains to be investigated. The severe pathology in the *slc12a2b^ue58^* mutant PNS also begs the question as to why CNS myelin is less severely affected. One possibility is that the second NKCC1-encoding gene in zebrafish, *slc12a2a,* can compensate for NKCC1b loss of function in the CNS, but not the PNS. Although we have not seen disruption to myelin in *slc12a2a* mutants (data not shown), the investigation of *slc12a2a, slc12a2b* double mutants will be required to test this. There is, however, only one NKCC1-encoding gene in mammals, and although mutant mice with conditional knockout of NKCC1 from the oligodendrocyte lineage show disrupted oligodendrocyte differentiation (Zonouzi et al., 2015), gross disruption to myelinated axon integrity was not reported. Therefore, an alternative explanation for the more severe effects of NKCC1b loss in the PNS may be the presence of factors with redundant functions in the CNS. For example, the inward-rectifying K^+^ channel Kir4.1 has been proposed to regulate ion homeostasis at the axon–myelin interface in the CNS (Larson et al., 2018; Schirmer et al., 2018), and so it would be interesting to test whether Kir4.1 and NKCC1 have redundant or distinct roles in maintaining myelinated axon integrity.

The mechanisms underpinning the complex physiology of myelinated axons, and in particular functional interactions at the axon–myelin interface and periaxonal space, remain to be fully elucidated. At present, there is increasing focus on the importance of the axon–myelin interface in the CNS, but our work indicates that this domain is also of key importance to peripheral nerves. Indeed, Schwann cells express numerous neurotransmitter receptors (Christensen et al., 2016; Chen et al., 2017), ion channels, and transporters (Baker, 2002), about whose functions in vivo we have much to learn. Understanding the interactions at the axon–myelin interface in both the PNS and CNS remains an important area of investigation to help fully elucidate myelinated axon formation, health, and function.

## Materials and methods

### Zebrafish husbandry and transgenic lines

Adult zebrafish were housed and maintained in accordance with standard procedures in the Queen’s Medical Research Institute zebrafish facility, University of Edinburgh. All experiments were performed in compliance with the UK Home Office, according to its regulations under project licenses 60/4035 and 70/8436. Adult zebrafish were subject to a 14-h/10-h light/dark cycle. Embryos were produced by pairwise matings and raised at 28.5°C in 10 mM Hepes-buffered E3 embryo medium or conditioned aquarium water with methylene blue. Embryos were staged according to dpf. The following lines were used in this study: Tg(mbp:EGFP-CAAX) (Almeida et al., 2011), Tg(cntn1b:mCherry) (Czopka et al., 2013), and Tg(claudinK:Gal4) (Münzel et al., 2012). The *ue58* allele was identified due to its striking disruption of mbp:EGFP-CAAX along the pLLn during the forward genetic screen, underpinning this study (described in Kegel et al., 2019; Klingseisen et al., 2019).

### Identification of genetic linkage and causative mutation

Following an outcross to WIK, pooled DNA from 116 *ue58* mutant recombinants was sequenced on an Illumina HiSeq4000 (Edinburgh Genomics). We processed this data through a modified version of the Variant Discovery Mapping CloudMap pipeline (Minevich et al., 2012) on an in-house Galaxy server using the Zv9/danRer7 genome and annotation. For both the Variant Discovery Mapping plots and assessing the list of candidate variants, we subtracted a list of wild-type variants compiled from sequencing of the *ekwill* strain plus previously published data (Butler et al., 2015; LaFave et al., 2014; Obholzer et al., 2012).

From the prospective candidate mutations in the region of chromosome 8 linked to the mutant phenotype, we filtered for prospective nonsense mutations likely to result in strong loss of function of encoded proteins. The candidate list was further filtered by excluding polymorphisms found in other species or other mutants that we sequenced that derived from the N-ethyl-N-nitrosourea (ENU)-based screen. We designed genotyping assays and identified only one candidate stop codon inducing mutation that was linked to the *ue58* mutant phenotype. This mutation resided in CABZ01084010.1 on chromosome 8 (Zv9) and was unique in all *ue58* sequence reads. From then on, to genotype *ue58* mutant animals, *ue58/+* heterozygotes and wild types, we amplified DNA surrounding the location of the mutation using the following primers: 5′-TGA​TGT​TTG​TGT​TTG​TTT​GGT​CTC​A-3′ and 5′-CGC​TCT​GAT​GGT​TTC​CTC​GG-3′.

The 145-bp wild-type PCR product is digested with MscI into 43-bp and 102-bp fragments, while mutant sequence remains uncut. Products were separated on a 2% agarose gel.

### Amplification of NKCC1-encoding ORF

Using the Basic Local Alignment Search Tool, we found alignment of sequence in the region of our candidate mutation with a separate, previously identified zebrafish gene, *slc12a2*, which encodes the solute transporter NKCC1.

To test whether a gene encoding a NKCC1-like product was encoded at the mutant-linked locus and to amplify full-length mRNA that might rescue the *ue58* mutant phenotype, we performed PCR with high-fidelity DNA polymerase Q5 (New England Biolabs [NEB]) from a pool of wild-type zebrafish total cDNA (reverse transcribed from total mRNA extracted from AB 5-dpf zebrafish). We used forward primer 5′-CAT​C**ATG**TCA​GAC​CAG​CCT-3′ (bases in bold denote start predicted codon) and reverse primer 5′-**CA**GGA​GTA​GAA​GGT​CAG​AAC-3′ (bases in bold denote first two bases of predicted stop codon), which were designed based on the partial transcript sequences available for each terminus of a possible *slc12a2b* gene. This PCR amplified a cDNA product of ∼3.2 kb, which we purified and TOPO cloned (using the Zero Blunt TOPO PCR Cloning Kit; ThermoFisher Scientific) to generate pCRII-slc12a2b. We sequenced four pCRII-slc12a2b clones, and in all, we identified a complete ORF of 3,276 bp. The termini-encoding regions of the ORF aligned well with the partial sequences in the database, and single-nucleotide variations were all annotated in SNPfisher (Butler et al., 2015) and similar between the clones, suggesting that these were true SNPs rather than mistakes introduced by the polymerase during PCR amplification. The *slc12a2b* cDNA was then subcloned into the pCS2+ vector for mRNA synthesis by digesting from pCRII-slc12a2b using EcoRI and ligating into EcoRI-digested and Alkaline Phosphatase, Calf Intestinal (CIP)-dephosphorylated pCS2+ vector. The *slc12a2b* cDNA sequence is available under NCBI accession no. MK648423.

For *slc12a2b^ue58^* mRNA rescue experiments, progeny from homozygous Tg(mbp:EGFP-CAAX), *slc12a2b^ue58^* parents were injected with 160 pg synthetic *slc12a2b^ue58^* mRNA at the one-cell stage and imaged at 4 dpf for quantification of mean myelinated nerve diameter compared with Tg(mbp:EGFP-CAAX) wild-type controls and *slc12a2b^ue58^* constitutive mutants.

### CRISPR-Cas9–based targeting of *slc12a2b*

To independently disrupt *slc12a2b* function, we used a CRISPR design tool (Integrated DNA Technologies) to identify guide RNA (gRNA) targeting sequences located in the putative exon 1 and putative exon 26 of the gene with predicted low off-target activity (exon 1, 5′-GGG​AAC​CCG​AGC​CAG​GCG​G-3′; exon 26, 5′-GGT​GGA​CAC​CGT​CCC​CTT​TC-3′). Cas9 protein (1 µg/µl final concentration; NEB) and gRNA (18 ng/µl final concentration) were mixed in Cas9 nuclease reaction buffer (20 mM Hepes, 100 mM NaCl, 5 mM MgCl_2_, and 0.1 mM EDTA, pH 6.5; NEB) containing 0.05% phenol red and incubated at 37°C for 10 min. Approximately 2–3 nl active gRNA-Cas9 ribonucleoprotein complex was injected into Tg(mbp:EGFP-CAAX) embryos at the one-cell stage and myelin morphology assessed in the days following injection. For each gRNA, at least two independent injection experiments were performed.

To genotype *slc12a2b^ue78^* homozygous mutant animals, *slc12a2b^ue78^/+* heterozygotes and wild types, we amplified DNA surrounding the location of the mutation in exon 1 using the following primers: 5′–GAA​GTT​CAC​CAC​ACG​GGA​CC-3′ and 5′-GAC​AAT​ACC​GGG​CGG​TGT​CC-3′. The 262-bp wild-type PCR product is digested with MwoI into 115-bp and 147-bp fragments, while the mutant sequence remains uncut. Products were separated on a 2% agarose gel.

### TEM

Control and *slc12a2b^ue58^* mutant animals were prepared at 4–5 dpf by high-pressure freezing using a Leica EM ICE apparatus (Leica Microsystems). As filler, a solution of 20% PVP in E3 embryo medium was used. Freeze substitution was performed as described (Weil et al., 2019). Epon-embedded animals were cut with a 35° diamond knife (Diatome) using a UC7 ultramicrotome (Leica Microsystems). Images were obtained with a LEO912 transmission electron microscope (Carl Zeiss Microscopy) equipped with a 2k on-axis charge-coupled device (CCD) camera (TRS) between 6,500 and 10,000× with the software iTEM version 5.2 (Olympus Soft Imaging Solutions). For overviews at higher magnification, 4 to 6 images were stitched to a multi-image assembly by the iTEM software. EM micrographs were processed using Photoshop Adobe Photoshop CS6 (13.0.1) x64 (Adobe Systems).

### Single-cell labeling

To mosaically label individual Schwann cells, we injected one-cell-stage Tg(claudinK:Gal4) embryos with a 1 nl solution containing 10 ng/µl pTol2-UAS:EGFP-pA or pTol2-UAS:memScarlet-pA to label the cytoplasm or membrane of control cells respectively along with 25 ng/µl *tol2* transposase mRNA. To analyze Schwann cells with disrupted *slc12a2b* function, we additionally injected embryos with CRISPR gRNA targeting exon 1 of the *slc12a2b* gene.

### Cell-type–specific targeting of *slc12a2b* in neurons and myelinating glial cells

To disrupt *slc12a2b* function specifically in neurons or myelinating glial cells, we cloned the highly efficient *slc12a2b* guide sequence targeting exon 1 into a Tol2 modular vector system that allows coexpression of Cas9 under a tissue-specific promoter (Ablain et al., 2015). Oligonucleotides encoding the 20 bp *slc12a2b* exon 1 guide sequence (Integrated DNA Technologies) were ligated into the pDestTol2CG2-U6:gRNA destination vector (Addgene) following BseRI (NEB) restriction digest under the zebrafish U6-3 promoter. This vector also contains GFP under the heart-specific *cmlc2* promoter as a marker of transgenesis. To enable neuron or glial-specific *slc12a2b* loss of function, we then performed Gateway reactions (Gateway Invitrogen) with 5′ entry vectors containing either a 5-kb genomic fragment of zebrafish neurofilament medium polypeptide a (nefma) regulatory sequence (see below) or a 6-kb fragment of neural-specific β tubulin (Kwan et al., 2007) or a 2-kb genomic fragment of zebrafish genomic *mbp* regulatory sequence (Almeida et al., 2011), with a middle entry vector containing membrane-bound tagRFPt, followed by the self-cleaving T2A peptide and zebrafish codon-optimized Cas9 sequence flanked by two nuclear localization signals and 3′ entry vector containing a polyA sequence (Kwan et al., 2007; Fig. 3 A).

One-cell-stage zebrafish embryos were injected with 1 nl of a solution containing 10 ng/µl plasmid DNA, 25 ng/µl transposase mRNA, and 0.05% phenol red. Embryos were screened at 3 dpf for transgene integration as indicated by green heart expression.

### Cloning of the nefma regulatory sequence

We amplified 5 kb of sequence immediately upstream of the nefma gene ORF (NM_001111214.2) from wild-type genomic zebrafish DNA using the following primers, which also included attB1 and attB2R sequences (bold) for cloning purposes: forward primer, 5′-**GGG​GAC​AAC​TTT​GTA​TAG​AAA​AGT​TG**CCA​CCG​TAA​TTA​ACA​AAT​ATC​CAT​CAC-3′; reverse primer, 5′-**GGG​GAC​TGC​TTT​TTT​GTA​CAA​ACT​TG**CGA​ACT​GAC​GGG​GAG​TGG​AGG​TG-3′.

The resulting PCR fragment was cloned into the pDONRP4-P1R plasmid to use as a p5E vector for gateway cloning.

### Pharmacological treatments

To inhibit neuronal electrical activity, we injected a 2-nl volume of 0.5 mM TTX (Tocris Bioscience) into the yolk of zebrafish larvae. A 3 mM stock of TTX, dissolved in water, was diluted in 10 mM Hepes-buffered E3 embryo medium (pH adjusted 7.4) containing 0.05% phenol red for injection. Control larvae were injected with a vehicle solution of E3 embryo medium containing 0.05% phenol red. For prevention experiments, 3 dpf larvae homozygous for *slc12a2b^ue58^*; Tg(mbp:EGFP-CAAX) animals and Tg(mbp:EGFP-CAAX), as controls, were used. For rescue experiments, either larvae homozygous for *slc12a2b^ue58^*; Tg(mbp:EGFP-CAAX) or one-cell-stage Tg(mbp:EGFP-CAAX) embryos injected with the construct mbp:memtagRFPt2Acas9; U6:Slc12a2b to enable glial-specific disruption of the gene *slc12a2b* were used. Larvae exhibiting signs of myelin pathology were imaged at 6 dpf and subsequently injected with TTX or a control solution followed by repeat imaging of the same region of the posterior later line (around the level of somite 6 for *slc12a2b^ue58^* mutants) 4–6 h later. The efficiency of injections was assessed by complete paralysis of larvae that persisted until the point of imaging. General health of injected larvae was assessed before imaging, and any larva showing signs of overt ill health were excluded from imaging and analysis.

### Live imaging and image analysis

For live imaging, zebrafish larvae were anaesthetized in 600 µM tricaine in embryo medium and mounted in 1.3% or 1.5% low-melting-point agarose. Live imaging of all transgenic reporters was performed on a Zeiss 880 LSM confocal microscope equipped with Airyscan, typically in superresolution mode, using a 20× objective lens (Zeiss Plan-Apochromat 20× dry, NA = 0.8). An Olympus microscope capable of DIC imaging was used to image tissue edema in *slc12a2b^ue58^* mutants using 60× water-immersion, NA = 1 objective lens. A Nomarski prism and polarizer were oriented in such a way as to provide DIC. All images depict a lateral view of the spinal cord with anterior to the left and dorsal to the top. Figure panels were prepared using Fiji and Adobe Illustrator CC version 24.1.1 (Adobe Systems).

To quantify myelin morphology from images of live Tg(mbp:EGFP-CAAX) animals, we performed automatic thresholding of maximum intensity projections, via the Huang method using ImageJ/Fiji (Schindelin et al., 2012, 2015). The thresholded images were then converted to masks and inverted, and objects were detected using ImageJ’s Analyze Particles function. Identified particles were then assessed for area (µm^2^) and relative fluorescence intensity (mean gray value), and total fluorescence was calculated as the sum of (mean gray value × particle area) for all relevant particles in any given image. The total visible myelinated nerve length was calculated (by summing all x coordinates uniquely occupied by particles) and used to calculate either the mean myelinated nerve or Schwann cell diameter (total area/visible myelinated nerve length).

### Statistical analysis

Statistical tests were performed using GraphPad Prism (version 8). Data were tested for normal distribution using D’Agostino–Pearson omnibus test and tested for significance by two-tailed Student’s *t* test or one-way ANOVA with Tukey’s multiple comparisons test where applicable. All data are expressed as mean ± SD. All data points represent individual animals unless otherwise specified as indicated in the figure legends, with symbols indicating the following P value ranges: *, P < 0.05; **, P < 0.01; ***, P < 0.001; ****, P < 0.0001.

### Online supplemental material

Fig. S1 shows that myelin in *ue58* mutants forms normally but becomes progressively disrupted. Fig. S2 shows a molecular characterization of the *ue58* mutation and *slc12a2b*. Fig. S3 shows that *slc12a2b* disruption leads to myelin pathology. Fig. S4 shows validation of *slc12a2b* loss of function in CRISPR/Cas9 mutant animals. Fig. S5 shows periaxonal space swelling, axonal enlargement, and myelin outfoldings in *slc12a2b^ue58^* mutants.
